# Therapeutic Effect of *Arsenicum album* on Leukocytes

**DOI:** 10.3390/ijms13033979

**Published:** 2012-03-22

**Authors:** Elaine C. Ive, Ingrid M. S. Couchman, Lalini Reddy

**Affiliations:** 1 Department of Homoeopathy, Faculty of Health Sciences, Durban University of Technology, P.O. Box 1334, Durban 4000, South Africa; E-Mails: elaine.ive0@gmail.com (E.C.I.); ingridc@dut.ac.za (I.M.S.C.); 2 Department of Biotechnology and Food Technology, Faculty of Applied Sciences, Durban University of Technology, P.O. Box 1334, Durban 4000, South Africa

**Keywords:** arsenic trioxide, *Arsenicum album*, homoeopathy, cell viability, hormesis

## Abstract

The therapeutic effects of homoeopathic *Arsenicum album* potencies were investigated *in-vitro*, using a continuous cell line (MT4), pre-intoxicated with arsenic trioxide (As_2_O_3_), and then treated with succussed and unsuccussed homoeopathic potencies, 6CH, 30CH and 200CH. This study aimed to verify the homoeopathic law of similars and to determine whether potencies diluted beyond Avogadro’s constant had physiological effects on cells; whether various potencies would cause different effects as suggested by the concept of hormesis; whether succussed and unsuccussed homoeopathic potencies had different effects on the cells; and to establish whether a biotechnological method could be used to evaluate the above. As_2_O_3_ was used to pre-intoxicate and the MTT assay was used to measure the percentage cytotoxicity and half maximal inhibitory concentration (IC_50_) of the cells. The homoeopathic potencies of *Arsenicum album* (6CH, 30CH and 200CH) were prepared by either succussing or allowing to diffuse for 30 s. After pre-intoxication of the MT4 cells with the IC_50_ As_2_O_3_ and treatment with succussed and unsuccussed *Arsenicum album* (6CH-200CH), the cell viability increased with increasing potency from 81% to 194% (over 72 h). The treatments and the times of exposure were found to be statistically significant determinants of cell viability, whereas succussion did not cause any significant variation in the results. The study provided evidence that a biotechnological method (namely cell viability) may be used to scientifically evaluate the physiological effects of homoeopathic potencies on human cells; it confirmed that the homoeopathic potencies did have therapeutic effects; and that succussion was not required in the potentization method in order to produce a curative remedy.

## 1. Introduction

Homeopathy is defined as a medical art that brings about cure through the administration of a medicine which is minute in dose and given according to similarity, by lifting the disease without pain or debilitation [1]. In order to evaluate the general principles of homoeopathy, an *in-vitro* study using human cells was designed, to determine whether the Law of Similars, hormesis, potentization and succussion could be reproduced on a cellular level. One of the fundamental principles in homoeopathy is the law of similars, also known as “Similia similibus curentur”, meaning “let like cure like” [1]. This study tested self-recovery of cells by applying a homoeopathic dose of the same substance that was responsible for the disease state; in this case *Arsenicum album* was given after the cells had been exposed to the IC_50_ As_2_O_3_ [2]. This was done according to the law of similars, where the potentization process caused the remedy to change slightly, and so was only similar and not identical to the original substance [1]. The phenomenon of hormesis is defined as a biphasic dose-response where stimulatory processes are induced with low doses [3]. Homoeopathic remedies are low concentrations that therefore stimulate, and lower potencies stimulate less than higher potencies [4]. This study tested and compared three different potencies of vastly different concentrations, in order to identify whether self-recovery of the cells would occur and which potency would stimulate self-recovery of the cells the most. Due to the concerns regarding whether potencies would have an effect when Avogadro’s constant was exceeded [5], this study aimed to further explore the concept of potentization and succussion beyond Avogadro’s constant by using 30CH (1 × 10^−60^) and 200CH (1 × 10^−400^) potencies, as well as 6CH (1 × 10^−12^) potencies where the original substance was still present. It is vital to identify an accepted scientific model of homoeopathic research that is reproducible, in order to strengthen the evidence of future experiments [6].

This study was based on the work done by Van Wijk [7] and Wiegant [8] who described how self-recovery at the organ level showed a shift of symptom picture over time; a toxin entered a system, damaged an organ, the organ began to regenerate and eliminated the toxin, which then affected a different organ and led to complications with a wide variety in the symptoms over time. It was noted that self-recovery at the cellular and molecular levels had no issue with the above time shift of symptoms [2]. The study did not however test potencies above Avogadro’s constant or compare the effects of succussed and unsuccussed potencies; these were therefore covered in this study, where high potencies were used to identify whether they had any effects on the cells, and succussed potencies were compared with unsuccussed potencies to identify the importance of succussion in remedy preparation.

Sukul, De, Dutta, Sukul and Sinhababu [9] compared succussed and unsuccussed 30CH Nux vomica preparations on toads to establish whether succussion was in fact an essential factor in the preparation of homoeopathic potencies. Their results showed that the toads responded to both the succussed and the unsuccussed potencies and that succussion was not an essential factor in producing an effective homoeopathic potency. However, Brizzi, Nani, Peruzzi and Betti [10] used a wheat germination *in-vitro* model and noted that succussed As_2_O_3_ potencies had significant and reproducible effects, whereas the unsuccussed As_2_O_3_ did not show any significant effects. These conflicting study results confirmed the necessity for further investigation into the need for succussion in homoeopathic potency preparation.

The MTT assay [11–14] measures the reduction of yellow-colored MTT (3-(4,5-dimethylthiazol-2-Yl)-2,5-diphenyltetrazolium bromide) to purple formazan crystals by the succinate tetrazolium reductase system which occurs in the mitochondria in viable cells in culture. Absorbance readings represent the amount of formazan dye which is directly proportional to the cell viability [15].

## 2. Results and Discussion

**Figure 1:**
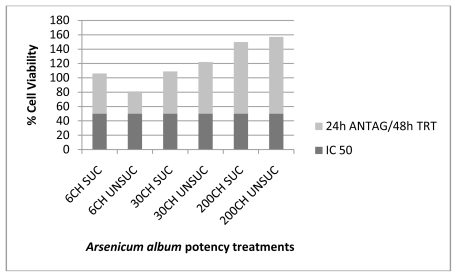
*Arsenicum album* treatment (48 h) of MT4 cells after 24 h antagonization with 5 μM As_2_O_3_.

**Figure 4:**
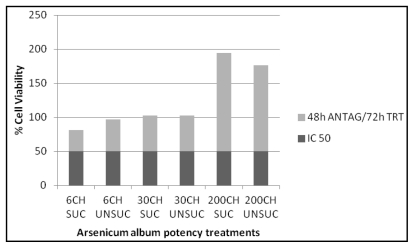
*Arsenicum album* treatment (72 h) of MT4 cells after 48 h antagonization with 5 μM As_2_O_3_.

The results obtained from this experiment (Table 1) verified the therapeutic effects of the various succussed and unsuccussed *Arsenicum album* potencies (6CH, 30CH and 200CH) on human cancer cells after intoxication with the IC_50_ of As_2_O_3_, by increasing the percentage of cell viability (Figure 1 to Figure 4). These results show absorbance as potency dependent; where each potency caused stimulation of the cells, increasing in amount as the potencies increased in dilution. It was also noted that the results for the succussed and unsuccussed potencies did not vary significantly.

The highest therapeutic effect was noticed thus when the MT4 cells were antagonized for 48 h with 5 μM As_2_O_3_ and then treated for 72 h with 200CH succussed *Arsenicum album* potency as shown in Figure 4.

SPSS version 15.0 was used to analyze the data. A *p* value of < 0.05 was considered as statistically significant. The effects of plate, succussion and treatment were examined with percentage cell viability as the dependent variable. It was found that the main effects of plate and treatment were statistically significant determinants of cell viability in the model (*p* < 0.001). There were no significant interaction effects, and succussion was also seen as not being significant (*p* > 0.05).

**Figure 5:**
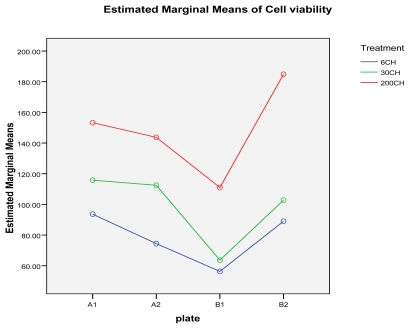
Estimated marginal means of cell viability for various *Arsenicum album* potencies (6–200CH) and exposure times (24 h, 48 h) on the MT4 cell line.

Figure 5 below represents the estimated marginal means of cell viability, where the succussed and unsuccussed potency results have been averaged; this shows that the 200CH with plate B2 provided the highest level of cell viability.

## 3. Experimental Section

Ethical approval for the use of human cell cultures was granted by the Faculty of Health Sciences Research Committee at the Durban University of Technology (DUT) in Durban, South Africa. This study was conducted in the cell culture laboratory in the Department of Biotechnology and Food Technology, at DUT, Durban, South Africa. The cytotoxic effects due to As_2_O_3_ exposure and the protective effects of succussed and unsuccussed *Arsenicum album* 6CH, 30CH and 200CH on the cells, were investigated by the MTT cell viability assay and trypan blue dye exclusion assay. All assays were performed in triplicate. Aseptic technique was used at all times whilst working with the cell cultures.

### 3.1. Culturing of the MT4 Cell Line

Passages 12 and 13 of the ATCC human T-cell lymphotropic virus type I-transformed T-cell line (MT4 cell line) were used in the experiments. The cells were in an actively growing state on arrival and were thus cultured in complete culture medium (CCM) immediately and incubated at 37 °C in a 5% CO_2_ humidified atmosphere. The CCM consisted of 35.8 mL of Roswell Park Memorial Institute (RPMI) medium, 4 mL (10%) filtered fetal calf serum (FCS), and was supplemented with 200 μL (0.5%) penicillin and streptomycin, Highveld Biological, South Africa [12].

### 3.2. Cell Enumeration Using the Trypan Blue Dye Exclusion Assay

50 μL of cell suspension was mixed with 50 μL of the 0.4% trypan blue solution, added to an eppendorf tube and vortexed. The viable (living) cells were impermeable to the trypan blue dye (Biowhittaker, Walkersville, MD, USA), therefore they appeared translucent, whereas the non-viable (dead) cells took up the dye and therefore stained blue.

### 3.3. The MTT Assay

MTT reagent was made up by adding 250 mg MTT (Sigma, USA) to 50 mL PBS, the tube was then covered with foil to protect the photo-sensitive MTT from the light, and stored at 4 °C overnight [16,17]. To each culture well of the 96-well plate (150 μL cell culture), 10 μL of MTT reagent was added. For the 24-well plate (1 mL cell culture), 67 μL of MTT reagent was added to each well. The plates were then placed on the shaker for 5 min in a dark room, followed by incubation for 3 h at 37 °C in 5% CO_2_ atmosphere. Next, 100 μL of DMSO was added to each well of the 96-well plate, and 667 μL of DMSO to each well of the 24-well plate. These were then placed on the shaker for 5 min in a dark room and then incubated for 25 min, to dissolve the formazan crystals. The amount of mitochondrial dehydrogenase present in the culture was read on the Cary 100 UV-visible spectrophotometer (Varian, Palo Alto, CA, USA), firstly by pipetting up and down each culture well to get the cells into suspension, and then by placing 1 mL of the culture in quartz cuvettes and reading at 578 nm. Filtered RPMI was used as the blank to initially zero the machine.

The percentage cell viability was determined using the formula:

$$\text{cell viability}=\frac{\text{Absorbance of treated cells}}{\text{Absorbance of}\;\text{untreated cells with solvent control}}\times 100\%$$

### 3.4. Solubilization of Arsenic Trioxide

Best solubilization of As_2_O_3_ was established by adding 396 mg dry As_2_O_3_ to 100 mL sterile distilled Milli-Q water. These were placed into a conical flask, completely sealed and positioned on a heating plate for 10 days at 80 °C, to aid in diffusion. This formed the As_2_O_3_ aqueous stock solution of 20 mM concentration. Any changes made to the arsenic solution in this process would not affect the experiment, as the antagonizer need only be similar and not identical to the remedy according to the Law of Similars.

### 3.5. Arsenic Trioxide Cytotoxicity

To define the correct dosage of As_2_O_3_ for the stress, the researcher tested different concentrations of As_2_O_3_ (1 μM, 5 μM and 10 μM), for different exposure times (24 h and 48 h) and used different injection volumes (5 μL, 10 μL, 20 μL and 50 μL), in order to identify the highest sub-lethal dose to cause 50% cell death. This was performed in triplicate using the MTT assay. 5 μM (133 μL) over 48 h was established as the optimum concentration of As_2_O_3_ to allow for 50% cell death (IC_50_).

### 3.6. Preparation of *Arsenicum album* Succussed and Unsuccussed Potencies

The *Arsenicum album* succussed (6CH, 30CH and 200CH) and unsuccussed (1 × 10^−12^, 1 × 10^−60^ and 1 × 10^−400^) potencies were prepared according to method 6 and 8b in the German Homoeopathic Pharmacopoeia (GHP) [18]. The remedies were first triturated and then, in sterile distilled water, either hand succussed 10 times or allowed to diffuse for 30 s, with the final potencies made up in RPMI (to prevent interference with cell cultures). Fifteen milliliters of each of the succussed and unsuccussed potencies were prepared.

### 3.7. *Arsenicum album* Potency Study Using the MT4 Cell Line

This experiment was performed so as to determine the therapeutic effects of the various *Arsenicum album* potencies (6CH, 30CH and 200CH) on cells after intoxication with the IC_50_ of As_2_O_3_, and to compare the effects of the succussed and unsuccussed potencies. The actively growing MT4 cells were diluted down to attain 2.45 × 10^5^ cells/mL; the number of viable cells was calculated by means of the trypan blue dye exclusion assay. Into two sterile 75 cm^3^ culture flasks, 50 mL of cell culture was added, each pre-intoxicated with 5 μM As_2_O_3_ (6.65 mL). The flasks were labeled A and B and incubated at 37 °C in 5% CO_2_ atmosphere for 24 h and 48 h respectively. After incubation, 8 mL of cell culture was decanted from each flask into a 15 mL centrifuge tube, which was then used later to plate cells untreated by potencies but containing As_2_O_3_. The remaining culture was then poured into a 50 mL centrifuge tube and centrifuged for 5 min at 3500 rpm. A pellet of cells formed at the bottom of the tube, and the media (containing As_2_O_3_) was carefully discarded. 50 mL of freshly made CCM was added to the pellet, which was then re-suspended. Into each well of the two 24-well plates, 1 mL of cell culture (2.35 × 10^5^ cells/mL—flask A; 2.25 × 10^5^ cells/mL—flask B) was added. Subsequently, 133 μL of the remedies, the solvent controls (RPMI) and negative controls (untreated) were added to each well and incubated for 48 h (plate A1 and B1) and 72 h (plate A2 and B2) respectively. After the prescribed incubation times, the MTT assay was performed on each plate in order to identify cell viability.

## 4. Conclusions

This study confirms the protection of the MT4 cells against the toxic effects of the As_2_O_3_ by the homoeopathic remedy, namely *Arsenicum album*; thereby verifying the Law of Similars. The results showed that the potencies diluted beyond Avogadro’s constant, namely the 30CH and 200CH, had physiological effects on cells, despite having no particles of the original substance present; and verified the theory of hormesis by confirming that different potencies have different effects on cells, with the more dilute potencies having the most stimulatory effects. This was observed where the 200CH caused the highest level of cell viability when compared to the 6CH, which caused the least. This study also showed comprehensively that succussion does not cause any significant change to the potency when testing on the cellular level. Finally, this study provided evidence that a biotechnological method could be used to evaluate the physiological effects of homoeopathic potencies on human cells.

## Figures and Tables

**Figure 2:**
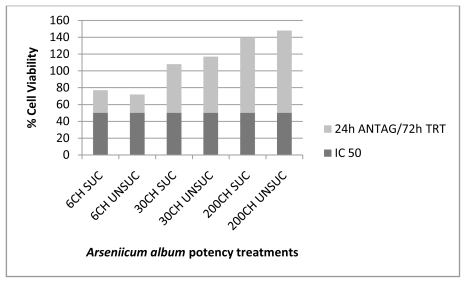
*Arsenicum album* treatment (72 h) of MT4 cells after 24 h antagonization with 5 μM As_2_O_3_.

**Figure 3:**
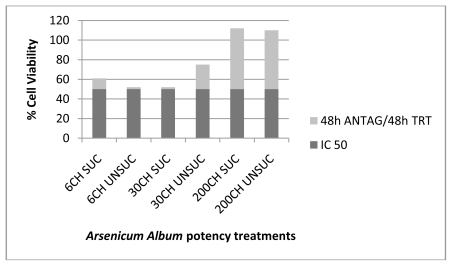
*Arsenicum album* treatment (48 h) of MT4 cells after 48 h antagonization with 5 μM As_2_O_3_.

**Table 1:** Absorbance values (578 nm) of *Arsenicum album* potency responses on MT4 cell line after intoxication with 5 μM As_2_O_3_.

		A1*		A2**		B1***		B2****	
	
		Mean	Standard Deviation	Mean	Standard Deviation	Mean	Standard Deviation	Mean	Standard Deviation
**Solvent control**		0.0306	0.0028	0.0240	0.0093	0.0332	0.0068	0.0378	0.0138
**6CH**	**Succussed**	0.0323	0.0019	0.0168	0.0004	0.0192	0.0023	0.0285	0.0029
	**Unsuccussed**	0.0249	0.0044	0.0175	0.0077	0.0178	0.0086	0.0349	0.0082
**30CH**	**Succussed**	0.0329	0.0072	0.0234	0.0014	0.0166	0.0025	0.0357	0.0018
	**Unsuccussed**	0.0376	0.0083	0.0268	0.0053	0.0242	0.0041	0.0373	0.0081
**200CH**	**Succussed**	0.0459	0.0038	0.0304	0.0043	0.0367	0.0134	0.0681	0.0035
	**Unsuccussed**	0.0476	0.0023	0.0333	0.0070	0.0361	0.0082	0.0609	0.0035

* Plate A1 = 24 h As_2_O_3_ followed by 48 h treatment;

** Plate A2 = 24 h As_2_O_3_ followed by 72 h treatment;

*** Plate B1 = 48 h As_2_O_3_ followed by 48 h treatment;

**** Plate B2 = 48 h As_2_O_3_ followed by 72 h treatment; 24 well plates (1 mL cell culture/well).
